# Fabrication of Microarrays for the Analysis of Serological Antibody Isotypes against Food Antigens

**DOI:** 10.3390/s19183893

**Published:** 2019-09-10

**Authors:** Jeahee Ryu, Soyoun Kim, Jaeseung Song, Daeun Kim, Narae Keum, Wonhee Jang, Hyosang Bae, Youngeun Kwon

**Affiliations:** 1 Department of Biomedical Engineering, Dongguk University, Seoul 04620, Korea; 2 Department of Life Science, Dongguk University, Seoul 04620, Korea; 3 Department of Sasang Constitutional Medicine, Dongguk University, Gyeongju 38066, Korea

**Keywords:** protein microarray, food intolerance, IgG, antibody isotypes, dietary pattern, IgE

## Abstract

Food intolerance is delayed adverse food reactions which follow consumption of specific foods. The underlying mechanisms are not well understood, but food intolerance is often considered as a type 2 hypersensitivity reaction mediated by immunoglobulin G (IgG) antibody. To understand the causes of food intolerance, it is important to investigate sensitization patterns of food-specific IgGs (sIgG) in relation to dietary patterns and physical conditions. Conventional approaches to measure serological IgGs often require large volumes of serum, thus are not suitable for highly multiplexed assays. To overcome this impracticality, we developed a highly sensitive method to screen the sIgGs and other antibody isotypes against 66 antigens with minimal amount of serums. We prepared a microarray by immobilizing food antigens on activated glass slides. Human sera and their dietary information were obtained from 30 subjects. Aliquots (200 nl) of sera were analyzed against 66 food antigens in parallel. sIgG levels were determined and analyzed in relation to subjects’ dietary patterns. The levels of antibody isotypes were also examined to understand the relationship between allergy and food intolerance. The developed microarray showed exceptional performances in antibody screening and demonstrated the potential to be used as an automated assay system.

## 1. Introduction

Adverse food reactions refer to any abnormal reactions occurring after consuming food and include food allergy and food intolerance. While there is a plethora of research on defining the mechanisms of allergic reactions, food intolerance has received less attention. Food intolerance is different from food allergy, type 1 hypersensitivity reaction, as it does not include traditional immunoglobulin (Ig) E-mediated reactions. Food intolerance is an unpleasant reaction to a specific food or food ingredient that can cause illness or discomfort in daily life. The symptoms associated with food intolerance include but are not limited to migraine [1], inflammatory bowel disease [2], irritable bowel syndrome (IBS) [3], dermatitis [4], and chronic constipation [5] and celiac disease [6]. Although these symptoms are not life-threatening, they can cause discomfort in daily life and lower the quality of life. The mechanisms and causes of food intolerance are not yet fully understood, but IgG has been suggested as a possible cause of food intolerance to classify the food intolerance as a type 2 hypersensitivity reaction. Several reports support this hypothesis. Hernandez et al. studied patients with recurrent refractory migraines to traditional pharmacological medications, noting statistically significant differences in the serological IgG levels and sensitization pattern between patients with migraines and the control group [7]. In the study, elimination diets successfully controlled the migraine without the need for medication. Shen and colleagues also reported that serological IgG antibodies against food antigens are distinctly elevated in patients with irritable bowel syndrome compared with healthy controls [8]. Zuo et al. demonstrated that patients with functional dyspepsia and irritable bowel syndrome have higher food-specific IgG levels, especially to crab, egg, shrimp, soybean, and wheat [9]. 

While these reports suggest that IgG may be the cause of food intolerance, there is insufficient data available on serological food-specific IgG levels in relation to dietary patterns or physical conditions. In order to study such a relationship, a systematic approach that utilizes a highly multiplexed assay format containing various food antigens as screening probes is necessary. Conventionally, serological food-specific IgGs were screened using immunoprecipitation, Western blot analysis [10], or enzyme-linked immunosorbent assay (ELISA) [11,12]. Among these, ELISA offers the most versatile platform for quantitative detection of specific IgGs (sIgGs) in multiplexed format. Vopli and Maccari showed that the ELISA is applicable to evaluate serum and plasma IgG response to food antigens in the Italian population [12]. While the ELISA-based assay is useful for detecting food-specific IgGs in human sera, this approach requires rather large volume of test samples when screening multiple targets. Microarray-based assays offer an improved alternative to these conventional approaches by presenting multiple antigens immobilized on the solid substrate allowing the simultaneous detection of multiple targets with improved sensitivity [13,14,15]. Currently, ImmunoCAP ISAC® is the most widely used microarray platform for allergy diagnostics. Although this allergy diagnostics platform could easily be converted to an IgG-screening platform, it is not suitable for the analysis of IgGs against food antigens since they mainly contain respiratory antigens, even further, in the form of component allergens. In addition, the food antigens presented in the platforms are mainly associated with the Western diet, which does not accurately represent the Korean diet.

In this work, we developed a microarray that can be used for screening food-specific IgGs in serum samples. The protein microarray was fabricated to represent common Korean diets for the analysis of serological IgG levels in relation to dietary patterns. The performance of the microarray-based assay platform was tested using standard IgGs. The limit of detection (LOD), as well as the reproducibility and accuracy of the microarray-based assays, were determined. We then screened 30 serum samples accompanied by dietary information using the microarray. We found that the levels of immune responses to specific food types are related to the frequency of its consumption and the characteristics of the food. Immunogenic and spicy food induced relatively high immune responses. Using this platform, we also investigated the correlation between IgE, IgG, and IgG4 to study the relationship between allergy and food intolerance. Finally, the microarray platform was tested for the analysis of whole blood samples to yield comparable results with the serum sample analysis.

## 2. Materials and Methods 

### 2.1. Materials

Tris and bovine serum albumin (BSA) were purchased from BioBasic (Markham, ON, Canada). Tween 20 was from Amresco (Solon, OH, USA). Rabbit anti-gliadin antibody was purchased from LS Bio (Seattle, WA, USA), and rabbit anti-ovalbumin antibody was purchased from Genetex (Irvine, CA, USA). Biotinylated goat anti-human IgG antibody (H+L), biotinylated goat anti-human IgE, and biotinylated goat anti-rabbit IgG was from VectorLab (Burlingame, CA, USA). Biotinylated mouse anti-human IgG4 and rabbit anti-casein antibody was purchased from AbCam (Cambridge, UK). Cy3-conjugated anti-human IgG was purchased from Jackson Immunoresearch (West Grove, PA, USA) and the other chemicals were purchased from Sigma-Aldrich (St. Louis, MO, USA) in their highest grades available, unless stated otherwise. Nexterion® Slide H was a product of Schott (Mainz, Germany). Antigen extracts of various sources were obtained from various sources; mugwort, rice, wheat flour, sweet potato, white potato, kidney bean, corn food, sesame, duck, chicken, pork, beef, oyster, shrimp, mackerel, salmon, cod fish, clam, ginger, crab meat, garlic, cucumber, lettuce, carrot, spinach, cabbage, mushroom, onion, watermelon, summer squash, raspberry, pineapple, peach, pear, grape, coffee, black tea, and cacao extract were purchased from Greer (Lenoir, NC, USA); gluten, barley flour, peanut, mung bean, buckwheat flour, honey, milk, egg, Cheddar cheese, abalone, whiting, anchovy, eel, tuna, seaweed, strawberry, banana, apple, mandarin, tomato, chestnut, pine nut, walnut, curry powder, green tea, arrow root, yam, chili pepper extract were bought from Squarix (Marl, Germany); and rice bran, glutinous rice, and adlay extract were donated from Wonmed (Bucheon, Korea).

### 2.2. Fabrication of Antigen Microarrays

An antigen microarray was constructed, by spotting antigen extracts onto a slide H using a microarrayer OmniGrid 100 from GeneMachines (San Carlos, CA, USA). Lyophilized antigens were first solubilized in distilled water to make 1 mg/mL solutions, while the dissolved antigens were used without further treatment. Phosphate-buffered saline (PBS), bovine serum albumin (BSA), and human serum albumin (HSA) were used as negative controls and biotinylated anti-human IgG was used as a positive control. Antigen solutions were mixed with the 10× PBS or 10× PBS containing 0.01% Tween 20 to make 1× PBS solution. The selected antigens and controls were spotted on a glass slide (Nexterion® slide H) coated with hydrophilic polymers and presenting NHS-ester, in triplicates. The microarrays were generated by spotting ~1 nl of antigens, in arrays of 200 μm diameter spots, at a spot-to-spot distance of 300 μm [16,17]. The size of an array was 5 × 10 mm or 6 × 5 mm. Four arrays were printed on each glass slide and separated using a 4-well hybridization chamber. After spotting, the antigen chips were stored at 4 °C, for at least 24 h, before use.

### 2.3. Immunoassay

The antigen microarray was treated with 2% BSA in PBST (PBS containing 0.1% Tween 20) for 1 h, washed with PBST, and incubated with 200 nl of human serum diluted to 1:800 in PBST for 1 h. The slide was washed with PBST, incubated with a solution of the detection antibody (biotinylated goat anti-human IgG, 1.5 μg/mL) in PBST containing 0.2% BSA for 1 h, and then treated with SA-Cy3 conjugate (1 μg/mL) in PBST containing 0.2% BSA for 15 min in dark. The slide was washed with PBST and distilled water, sequentially, and then, dried using compressed air. All steps were carried out at room temperature. For the whole blood sample analysis by the immunoassay procedure, the whole blood was first treated with 5 mM EDTA to prevent coagulation and was diluted to 1:800 with PBST. Detection of serological IgE was performed using previously described procedure [18]. A model microarray presenting protein L was fabricated using various concentrations of protein L from 0.01 to 1 mg/mL. The bound human IgGs were visualized using Cy3-labeled goat anti-human IgG antibody.

### 2.4. Scanning

The Cy3 fluorescence intensity was measured by using a GenePix 4000B fluorescence scanner from Axon Instruments (Union City, CA, USA) at an excitation wavelength of 532 nm and an emission wavelength of 570 nm. The image files were analyzed using GenePix Pro Microarray Analysis software. Three intensity values for each antigen, were obtained from triplicates. The average fluorescence intensity of the empty spots was subtracted from the measured values, thereby providing the background signal for calibration. The average fluorescence intensity was calculated and used for the analysis.

### 2.5. Serum Samples and Food Sensitivity Testing

This research was approved by the Institutional Review Boards of Dongguk University Ilsan Oriental Hospital (IRB number DUIOH 2017-01-002-002) on July 4, 2017. Serum collection started from July 12, 2017. Healthy women aged between 25 and 45 years were screened. In order to minimize confounding factors, subjects who were currently pregnant, had given birth within 2 years, and were diagnosed with hypertension were eliminated. Subjects taking diabetes medication, steroid medication, oral contraception, or immune disease medications were also excluded. Finally, patients with surgery records within 1 year were eliminated. From 30 subjects, we collected self-answer questionnaires of their daily food intake patterns and serum samples, which were screened for IgE and IgG reaction against the selected antigens, and the results were subjected to statistical analysis.

### 2.6. Statistical Analysis

All statistics were performed using R, and the significance was defined as *p* < 0.05.

## 3. Results and Discussion

### 3.1. Fabrication and Evaluation of a Model Microarray

A model microarray presenting ovalbumin, casein, and gluten in a series of concentrations from 0.03 to 0.2 mg/mL was prepared using SCHOTT Nexterion® Slide H. Slide H is a glass slide coated with a crosslinked organic hydrogel activated with highly reactive *N*-hydroxysuccinimide (NHS) esters to allow covalent immobilization of amine groups while the uniquely constructed coating matrix inhibits nonspecific binding of proteins. Slide H provides an excellent signal-to-noise ratio in microarray experiments [18]. 

**Figure 1:**
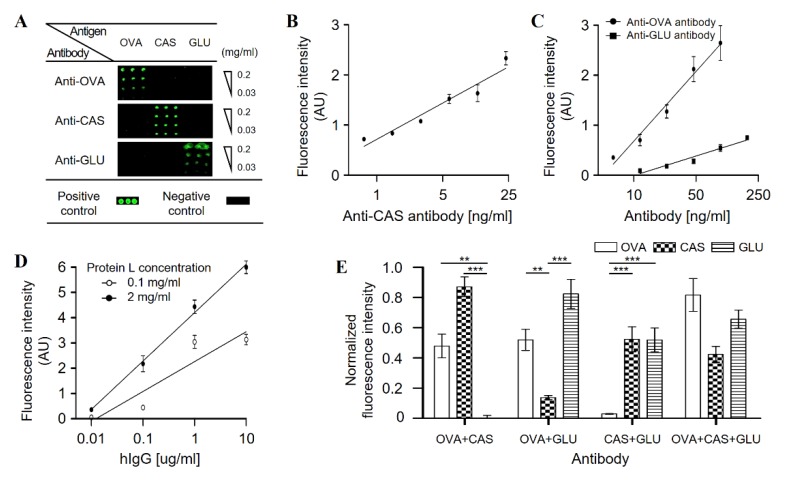
Fabrication of model microarray and performance evaluation (**A**) A model microarray presenting ovalbumin, casein, and gluten was prepared and used to screen specific antibodies, anti-ovalbumin antibody, anti-casein antibody, or anti-gluten antibody, individually. (**B**) The fluorescence intensity showed a linear concentration dependence for anti-CAS antibody. (**C**) The fluorescence intensity showed a linear concentration dependence for anti-OVA and anti-GLU antibody. (**D**) Human IgGs were quantitatively analyzed using the model microarray, presenting protein L in different concentrations. (**E**) Multiple target detection by the model microarray using different combinations of sIgGs. ** indicates *p* < 0.01 and *** indicates *p* < 0.001 using two-way ANOVA analysis. OVA: ovalbumin, CAS: casein, GLU: gluten, hIgG: human immunoglobulin G, sIgG: specific immunoglobin G.

The performance of the microarray was evaluated by measuring the sensitivity and specificity using standard antibodies. The assays were performed as previously described [18]. In brief, antigen-spotted microarray was blocked with 2% BSA in PBST buffer. Each corresponding standard rabbit IgGs against ovalbumin, casein, or gluten was treated in varying concentrations from 0.18 to 375 ng/mL. Then the microarray was treated with biotinylated goat anti-rabbit IgG and fluorescently labelled streptavidin in sequence, for quantitative analysis of bound standard IgGs. Fluorescent signal intensity of each spot was analyzed by a laser scanner. (Figure 1A) The fluorescence intensity was plotted against the concentration of antibodies, showing linear responses within the range of concentrations tested. (Figure 1B,C). LOD was calculated using the following equation [19]:

LOD=3*S*/*b*

where *S* is the standard deviation of the y-residuals; and b is the slope of the calibration curve. The calculated LOD was 2.73 ng/mL when determined with anti-casein IgG.

Since human sera contain a myriad of antibodies in various types, good specificity of the screening assay is a critical factor in evaluating the performance of the screening platform. The specificity of the developed food microarray platform was assessed by detecting multiple targets simultaneously while discriminating non-target antigens using combinations of two model antibodies. Two different antibodies were mixed in various ratios to a final concentration of 10 ng/mL to prepared test samples. The assays were performed using the test samples on model microarrays. The normalized fluorescence intensity of each antigen spot was then plotted for comparison. Each combination of antibodies showed expected results by detecting target antigens while discriminating non-target antigens (Figure 1E). This result showed that the developed food microarray platform had adequate performance for detecting the sIgGs of interest. 

We also prepared another model microarray presenting protein L to determine the concentration of total serological IgGs in each sample, as protein L binds to various antibody isotypes with high affinities [20]. Protein L was spotted in concentrations ranging from 0.01 to 2 ng in triplicates. The assays were performed using standard IgGs in known concentrations and the binding of standard IgG was visualized using fluorescently-labeled goat anti-human IgG as a secondary IgG. The plot of fluorescence intensities versus IgG concentrations was obtained and used as a standard curve to determine the concentration of serological IgGs (Figure 1D). The total concentration of serological IgG was 9.98 mg/mL which was well within the range of reported values of 4.07 to 21.7 mg/mL [21].

### 3.2. Fabrication and Evaluation of Food Microarray

**Figure 2:**
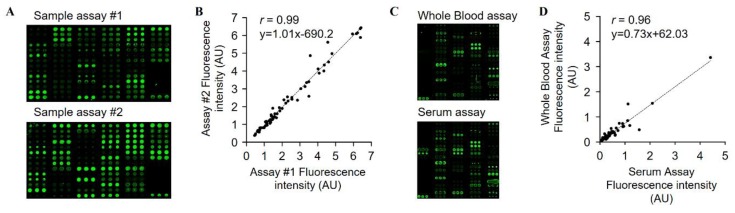
Fabrication of food microarray and performance evaluation (**A**) Two serum samples obtained from two different human subjects showed discrete sensitization patterns. (**B**) The same serum sample was analyzed using two replicate arrays, assay #1 and #2, to test the reproducibility of the microarray-based assays. The two independent assays showed an excellent correlation with Pearson’s *r* of 0.99. (**C**) Whole blood and serum obtained from the same human subject showed similar sensitization pattern. (**D**) The whole blood and the serum samples obtained from the same subject were analyzed individually. The whole blood sample showed decreased signal intensity, but both assays showed an excellent correlation with Pearson’s *r* of 0.96.

We selected 66 food antigens based on common dietary patterns in the Korean population [22]. The antigens were selected from seven different food categories: grains, vegetables, fruits, dairy products, meats, seafood, and miscellaneous (Table 1). Several antigens commonly known to induce food intolerance or allergies, such as milk, egg, shrimp, crab, pork, gluten, pineapple, soybean, and peanut, were also included. The food antigens, i.e., food protein extracts, were spotted in triplicates in 14 by 6 arrays (5 × 10 mm) or 20 by 4 arrays (6 × 5 mm) on Slide H. Each array included internal controls; BSA, HSA, PBS as negative controls and biotinylated goat anti-human IgG antibody as a positive control. The autofluorescence of the spotted antigens were negligible (data not shown). The assays were performed using serum samples, biotinylated anti-human IgG antibody, and fluorescently-labelled streptavidin, in sequence, as described above (Figure 2). 

First, the reproducibility of the microarray-based assay was determined by comparing the results of two independent assays using the same serum sample. The amount of sIgGs produced in response to 66 different antigens were plotted on a graph. The plots showed an excellent correlation with a Pearson’s correlation (*r*) value of 0.99 (Figure 2B) showing that this microarray-based assay had outstanding reproducibility. We then determined the intra-assay and inter-assay variation for eight different antigens to assess the accuracy of the assay (Table 2). The intra-assay variability, calculated using the variation between triplicated spots in one single array, was 6.6~17.1%. Notably, the intra-assay variability was smaller when the antigen spots were near-perfect circle, whereas irregular shaped spots, for example, spread (chili pepper, rice, and gluten) or unorthodox half-moon patterns (pork), showed higher intra-assay variability. The inter-assay variability from three independent arrays, was 1.2~10.5%. Six out of eight antigens gave inter-assay variability values smaller than 5% and two irregular-shaped antigen spots, namely chili pepper and rice, gave an inter-assay variability of 9.2 and 10.5%, respectively. Both the intra-assay and inter-assay variations were smaller than the previously described microarray-based assays [23]. These collective evaluation results suggested that the developed microarray enables precise and accurate results in screening sIgGs and allows quantitative analysis of serological IgGs.

### 3.3. Detection of Serological IgG Using Microarray and Data Analysis in Comparison to Dietary Information

**Figure 3:**
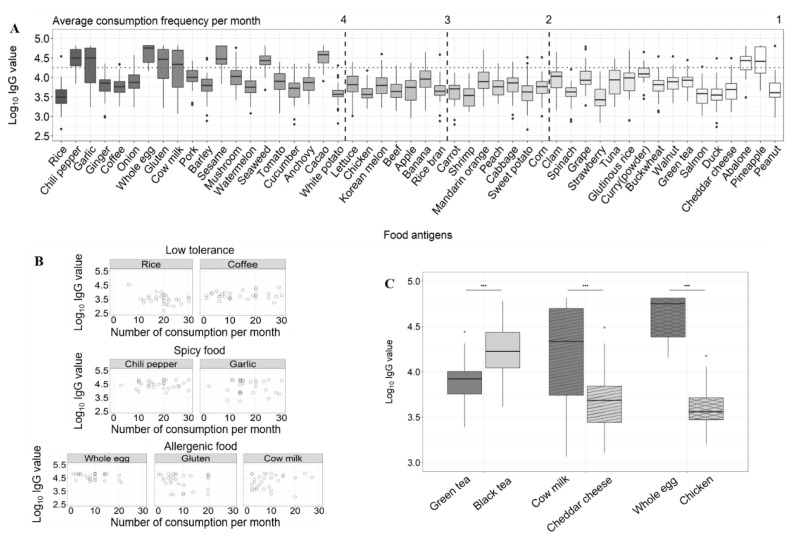
Analysis of serological IgG levels in comparison to dietary patterns of subjects (**A**) Correlation between the average consumption frequency per month and sIgG values. Foods on the x-axis were aligned in descending order of consumption, and sIgG values on the y-axis were transformed to log_10_ IgG values. (**B**) Correlation between consumption frequencies and IgG values in selected groups. Selected foods belonging to three distinctive food groups, namely low tolerance, spicy, and allergenic foods, were presented. (**C**) Comparison of foods with the same origin. Statistical analyses were conducted with two-tailed Wilcox test. *** indicates *p* < 0.001. IgG: immunoglobulin G, sIgG: specific immunoglobin G.

We collected human sera from 30 subjects along with their dietary information. The analysis of the dietary information showed that rice was the most highly consumed food (19.8 times/month on average), followed by chili pepper (17.9 times/month on average), garlic (16.9 times/month on average), ginger (16.5 times/month on average), and then coffee (14.1 times per/month on average) in the Korean diet (Figure 3A). Some food antigens were rarely consumed during the study duration, such as oyster, which is seasonal food. We analyzed the titer of sIgGs to food antigens that were consumed at least once per month on average.

The collected sera were analyzed using the microarray-based assay to detect the sIgGs against 66 food antigens. The screened sera of two different test subjects showed discrete sensitized patterns (Figure 2A). The high median values indicated elevated levels of sIgGs, whereas the wide distribution range suggested mixed population of individuals with different levels of sIgGs. We made three distinctive observations from the relationship between the sIgGs levels and certain properties of the food groups in the dietary patterns (Figure 3B). First, more frequently consumed food groups had higher median sIgG values compared with less frequently consumed food except some food antigens, such as rice and coffee that had very low median sIgGs values even though they were highly consumed. These foods could be categorized as non-stimulating. A previous study by Zuo *et al.* also demonstrated that patients with IBS had comparable serum IgG titers to rice as the healthy controls, suggesting low immunogenicity of rice [9]. Second, we paid attention to naturally immunogenic food groups, food allergens that were consumed more than four times a month such as whole egg (9.1 times/month on average), gluten (9.0 times/month on average), and cow milk (8.9 times/month on average). These food antigens also had very high median and wide-ranging sIgG values compared with the other food groups. Notably, infrequently consumed food allergens, such as abalone and pineapple also showed an unexpectedly high median and broad-ranging sIgG values. This observation probably suggests that the sIgG levels for immunogenic food groups are higher than non-immunogenic food groups, overall. Our third finding was that spicy foods, such as chili pepper and garlic, also had high median IgG values. Even though chili pepper and garlic are known to cause dietary discomfort, such as diarrhea and stomach pain, they are the second and the third most frequently consumed foods, respectively, in Korea, probably because these two foods are present in almost all Korean cuisine and so cannot be easily eliminated from the diet. 

We then compared the sIgGs levels of the food antigens from the same or related sources; black tea was fermented from green tea, Cheddar cheese from milk, and chicken developed from egg (Figure 3C). Black tea had significantly elevated median sIgG levels compared with green tea (Mann-Whitney test, *p* < 0.001). Cheddar cheese had a significantly lower median sIgG level and smaller variation in comparison to milk (Mann-Whitney test, *p* < 0.001). Whole egg had a significantly higher level of median sIgG and wider variation compared with chicken (Mann-Whitney test, *p* < 0.001). Together, these results show that foods from the same source have very different immunogenic reactions perhaps because of the change in the protein and carbohydrate composition during fermentation or development. Black tea and green tea are produced from the leaves of *Camellia sinesis*. During the fermentation of black tea, theaflavins, thearubigins, and other components are produced that are rarely present in green tea [24]. When comparing cheese and milk, milk showed a higher sIgG level, this may be explained by the presence of lactose, which is the major component that causes food intolerance in cow milk, but which is removed during the fermenting process. Whole egg contains very few proteins compared with chicken; however, the major component of egg that causes food intolerance, namely ovalbumin, is largely removed during development. Overall, the data suggested that there were mixed populations of subjects with/without food intolerance to milk and egg and that the removal of the components responsible for causing the food intolerance significantly reduced the median sIgG values.

### 3.4. Correlation Analysis of IgE, IgG, and IgG4

**Figure 4:**
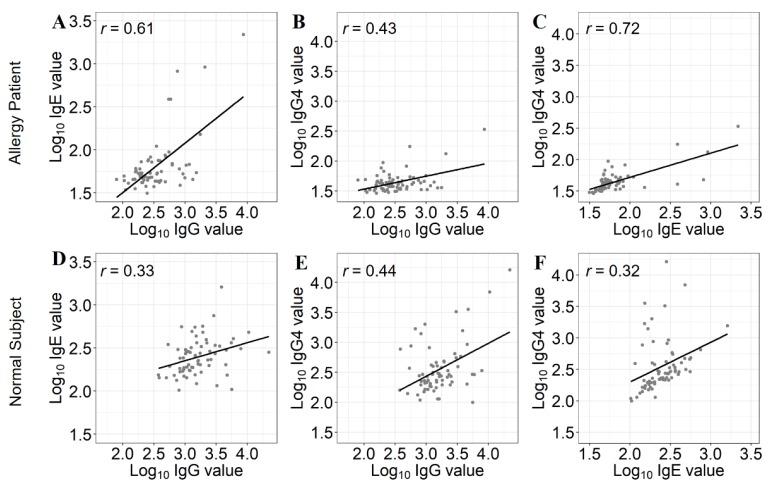
Correlation of serological immunoglobulin isotypes in allergic patient and control subject. Upper panel (**A**–**C**) represents the data obtained from allergic patient. (**A**) Correlation between IgG and IgE. (**B**) Correlation between IgG and IgG4. (**C**) Correlation between IgE and IgG4. Lower panel (**D**–**F**) indicates the data obtained from non-allergic control subject. (**D**) Correlation between IgG and IgE. (**E**) Correlation between IgG and IgG4. (**F**) Correlation between IgE and IgG4. Pearson’s correlation coefficients were shown inside the graphs. Ig: immunoglobulin.

The developed microarray-based assay platform enables simultaneous monitoring of various isotypes of antibodies. Thus, we investigated the similarity of the antigen specificity pattern between serological IgG, IgE and IgG4 using the microarray. We intended to obtain information on the correlation between allergies and food intolerance. It has been reported that IgE and IgG4 are the most prominent isotypes in human response to allergens [25]. We compared the levels of IgE, IgG, and IgG4 in the sera of an allergic patient and a non-allergic (control) subject. The patient was sensitized to crab and shrimp, whereas that of control subject was not. The microarray-based assays were performed as described above, by using secondary antibodies against human IgE, human IgG, and human IgG4 for detecting IgE, IgG, and IgG4, respectively. The signal intensities of sIgE, sIgG4, and sIgG were normalized and compared to determine the correlation between the antibody levels (Figure 4A–F). We observed that the patient’s serum showed high sIgE titers to crab and shrimp, as expected (data not shown). In order to analyze the data, we performed Pearson’s correction analysis. In both cases, the correlations between IgG and IgG4 were moderately strong with R values of 0.44 (Figure 4B,E), as expected since we measured total IgGs including the IgG4 isotype. The correlation of IgG with IgE as well as IgG4 with IgE were both much higher for the patient’s serum than the control subject’s serum. Pearson *r*-values were 0.62 and 0.72 for IgG/IgE and IgG4/IgE correlation analysis, respectively, when the assays were performed using the patient’s serum (Figure 4A,C). When the assay was performed using the control subject’s serum, smaller Pearson *r*-values of 0.33 and 0.32 for IgG/IgE and IgG4/IgE correlation analysis, respectively, were obtained (Figure 4D,E). The results showed that serological IgG and IgE as well as IgG4 and IgE titers of an allergic patient had a significantly stronger positive correlation compared with the control subject (Fisher’s Z transformation, *p* < 0.05). While the mechanisms of IgGs involvement in allergic responses is still unclear, our finding is in accordance with previous reports suggesting that the levels of IgG4 increased with the increase in IgE in the allergic patients’ sera [26,27]. 

### 3.5. Analysis of Whole Blood Using Microarray

Many immunoassays are performed using human sera instead of whole blood because whole blood contains various components that can interfere with the assays, such as fibrinogen, peripheral blood mononuclear cells, red blood cells, and platelets. However, separation of serum from the whole blood requires an extra pre-assay preparation step, which makes it difficult to automate the microarray-based assay. In order to address this issue, we determined the serological antibodies titers using a fresh whole blood sample and compared that with the results obtained using the serum sample. The serum and whole blood samples were obtained from the same subject and analyzed using two replicate arrays on one slide to minimize the batch effect. The assay result was analyzed by comparing the signal intensities of the two assays using Pearson’s correlation analysis. Pearson’s *r*-value was 0.96, showing that the two assays using serum and whole blood were highly comparable (Figure 2C,D). The correlation plot of fluorescence intensity presented a linear correlation with a slope value of 0.73, indicating that the signal intensity of the assay using serum was slightly higher than that of whole blood. This difference, albeit small, might be due to the presence of various interfering components in whole blood. The data suggest that our assay can successfully be performed using a whole blood sample and circumvent the pre-assay separation step. The capability to analyze the whole blood sample instead of serum samples is critical in developing a self-diagnostics kit.

## 4. Conclusions

In this work, we developed microarrays using food antigens for monitoring serological antibody isotypes related to food intolerance and food allergies. The microarray contained 66 food antigens representatives of the Korean dietary pattern to study the relationship between dietary patterns and food-specific antibody levels in the Korean population. The developed microarray showed outstanding performance with excellent specificity and sensitivity. The LOD was 2.73 ng/mL outperforming the gold standard assay based on ImmunoCAP® (measuring range: 0.002–0.2 mg/mL) [28]. By analyzing the sIgG level in the context of the dietary pattern, we observed that more frequently consumed food groups, food groups that cause food intolerance, and spicy foods had high median IgG levels. In addition, certain food types with the same or similar origins, such as black tea and green tea, Cheddar cheese and milk, and chicken and egg, had significantly different (*p* < 0.001) median IgG values probably due to the variation in the protein and carbohydrates compositions during the fermentation or development. It needs to be noted that the data used in this study were collected by a survey, and we could not control the frequency of food consumption to further validate our hypothesis. The assays were implemented to detect three different serological antibody isotypes, IgE, IgG, and IgG4, using the samples from an allergic patient and a non-allergic subject. The correlation analysis between the antibody isotypes revealed a stronger positive correlation of IgE with IgG and with IgG4, obtained from the serum of the allergic patient than the non-allergic subject. These results suggested that not only IgE but also IgG isotypes are involved in human allergic responses. Finally, the developed microarray platform showed a satisfactory performance in the analysis of the whole blood samples highlighting its capability for automation, as well as for the development of a self-diagnostics kit.

## Figures and Tables

**Table 1:** Food antigens presented on the microarray.

Categories	Food Antigens
**Grain**	Mung Beans, Buckwheat, Barley, Rice, Rice bran, Corn, Adlay extract, Sesame, Glutinous rice
**Vegetable**	White potato, Sweet potato, Chili pepper, Carrot, Peanut *, Yam, Mushroom, Lettuce, Spinach, Mugwort*, Cabbage, Onion, Cucumber, Tomato, Summer squash,
**Seafood**	Crab*, Big mackerel*, Oyster, Seaweed, Codfish, Anchovy, Whiting, Shrimp *, Salmon, Eel, Abalone, Clam, Tuna
**Fruit**	Mandarin orange, Strawberry, Banana, Chestnut, Pear, Peach, Apple, Watermelon, Pine nut, Pineapple, Grape, white, Walnut
**Meat/Dairy Product**	Whole egg, Cow milk *, Cheddar cheese, Chicken, Pork, Beef, Duck
**Others**	Gluten, Green tea, Garlic, Honey, Ginger, Arrow root, Curry, Cacao, Coffee, Black tea

* These antigens are previously analyzed on microarray for serological IgE detection [18].

**Table 2:** Intra- and inter-assay variability determined using the developed microarray platform for selected food antigens.

Antigen	Milk (Cow)	Egg Whole	Chili Pepper	Rice	Gluten	Garlic	Pork	Cacao
Intra-assay Variability (%)	6.7	6.6	10.3	17.1	9.9	11.6	16.0	14.6
Inter-assay Variability (%)	2.5	1.2	9.2	10.5	3.9	3.9	4.7	4.1
